# Genome-wide association studies of dairy cattle resistance to digital dermatitis recorded at four distinct lactation stages

**DOI:** 10.1038/s41598-025-92162-x

**Published:** 2025-03-15

**Authors:** Eirini Tarsani, Bingjie Li, Alkiviadis Anagnostopoulos, Matthew Barden, Bethany E. Griffiths, Cherry Bedford, Mike Coffey, Androniki Psifidi, Georgios Oikonomou, Georgios Banos

**Affiliations:** 1 Department of Animal and Veterinary Sciences, Scotland’s Rural College (SRUC), Easter Bush, Midlothian, EH25 9RG UK; 2 Department of Livestock and One Health, Institute of Infection, Veterinary and Ecological Sciences, University of Liverpool, Leahurst Campus, Neston, CH64 7TE UK; 3 Royal Veterinary College, Hawkshead Lane, Hatfield, Hertfordshire AL9 7TA UK

**Keywords:** Digital dermatitis, Dairy cattle, Genome-wide association study, Genetic association study, Genome-wide association studies

## Abstract

Digital dermatitis (DD) is an endemic infectious hoof disease causing lameness in dairy cattle. The aim of the present study was to investigate the genetic profile of DD development using phenotypic and genotypic data on 2192 Holstein cows. The feet of each cow were clinically examined four times: pre-calving, shortly after calving, near peak of milk production, and in late lactation. Presence or absence of disease and proportion of healthy feet per cow constituted two DD phenotypes of study. For each phenotype and timepoint of clinical examination, we conducted single-step genome-wide association analyses to identify individual markers and genomic regions linked to DD. We focused on the ten 1-Mb windows that explained the largest proportion of the total genetic variance as well as windows that enclosed significant markers. Functional enrichment analysis was also applied to determine functional candidate genes for DD. Significant (*P* < 0.05) genomic heritability estimates were derived ranging from 0.21 to 0.25. Results revealed two markers on chromosomes 7 and 15 that were related to both disease phenotypes. Furthermore, we identified three genomic windows on chromosome 14 and one window on chromosome 7 each explaining more than 1% of the trait additive genetic variance. Functional enrichment analysis revealed multiple promising candidate genes implicated in hoof health, wound healing, and inflammatory skin diseases. Collectively, our results provide novel insights into the biological mechanism of host resistance to DD development in dairy cattle and support genomic selection towards improving foot health.

**Supplementary Information:**

The online version contains supplementary material available at 10.1038/s41598-025-92162-x.

## Introduction

Digital Dermatitis (DD) is an infectious painful, contagious, superficial ulcerative condition affecting the hoof skin in animals of ruminant species^1^, typically resulting in clinical lameness^2^ and decreased productive and reproductive efficiency^3^. DD affects animal welfare^2,4,5^ and causes severe lameness in cattle^2^ and sheep^6^ worldwide. The disease is difficult to manage as it is persistent and has different clinical manifestations and a high rate of recurrence after therapy^3^. The economic impact of DD due to reduced milk production, discarded milk, treatment costs, and additional labour ranges from $64.00^7^ to $132.96 per disease episode^8^. A previous UK study reported the cost of individual DD lesions at £75.57^9^.

The aetiopathogenesis of the disease is not fully understood but therapeutic interventions as well as microbiological, immunological, ultrastructural, and molecular evidence indicate that *Treponema spp.* play an important role in the multifactorial pathogenesis of DD lesions^2–4^*.* New strategies to control the transmission of DD are needed, as no efficient vaccine exists to date and the application of (topical) antibiotics and chemicals in footbaths may induce bacterial resistance^3^. Previous studies^10,11^ have demonstrated the presence of genetic variation in animal susceptibility to different manifestations of DD infection, supporting the possibility to enhance the inherent resistance capacity of the cows as a complementary means to controlling the disease. A better knowledge of the genetic variation and the genetic basis of DD resistance provides essential information for future genetic improvement of disease resistance of DD in dairy cattle.

The present study aims to contribute to an enhanced understanding of the genetic architecture of dairy DD resistance using a relatively large population of Holstein dairy cows in the UK. We examined two disease status phenotypes based on foot examination at four distinct stages of the lactation cycle, each associated with different levels of milk production and representing varying metabolic and physiological profiles and challenges for the cow. We also conducted single-step genome-wide association studies to identify individual markers and genomic regions associated with each disease phenotype. In addition, functional enrichment analysis was applied to reveal plausible candidate genes for DD.

## Results

### Genomic parameters

The heritability estimates for the phenotype denoting presence or absence of DD (BINP) were 0.24 (SE = 0.02), 0.22 (SE = 0.02), 0.23 (SE = 0.02) and 0.21 (SE = 0.03) at pre-calving (DRY; 0–120 days before calving, mean = 55.3 days before calving), immediately after calving (FRESH; 0–21 days in milk, mean = 5.4 days in milk), near peak milk yield (PEAK; 50–120 days in milk, mean = 83.9 days in milk), and the latter part of lactation (LATE; 170–305 days in milk, mean = 199.6 days in milk), respectively. For the trait describing the proportion of healthy feet per cow (PROP), corresponding estimates were equal to 0.25 (SE = 0.02), 0.21 (SE = 0.02), 0.23 (SE = 0.02) and 0.23 (SE = 0.03), respectively. Genetic correlations between timepoints of foot clinical examination are summarised in Table 1 and ranged from 0.88 (SE = 0.04) to 0.99(SE = 0.04).

**Table 1:** Genetic correlation estimates (± standard errors) between timepoints* of foot examination for presence/absence of digital dermatitis (BINP; above the diagonal) and proportion of healthy feet (PROP, below the diagonal).

	DRY	FRESH	PEAK	LATE
DRY		0.95 ± 0.03	0.88 ± 0.04	0.90 ± 0.05
FRESH	0.94 ± 0.03		0.99 ± 0.03	0.99 ± 0.04
PEAK	0.92 ± 0.04	0.91 ± 0.04		0.97 ± 0.04
LATE	0.91 ± 0.04	0.90 ± 0.05	0.97 ± 0.03	

*DRY: up to 120 days before calving, FRESH: 1–21 days after calving, PEAK: 50–120 days after calving and LATE: 170-305 days after calving.

### Single-locus analysis

The Q–Q plots and the genomic inflation factor (λ) estimates from the genomic analysis of BINP at each timepoint are given in Supplementary Fig. 1. The λ estimates ranged from 0.99 to 1. These results clearly show no evidence of systematic bias attributed to population structure or analytical approach^12^.

**Fig. 1:**
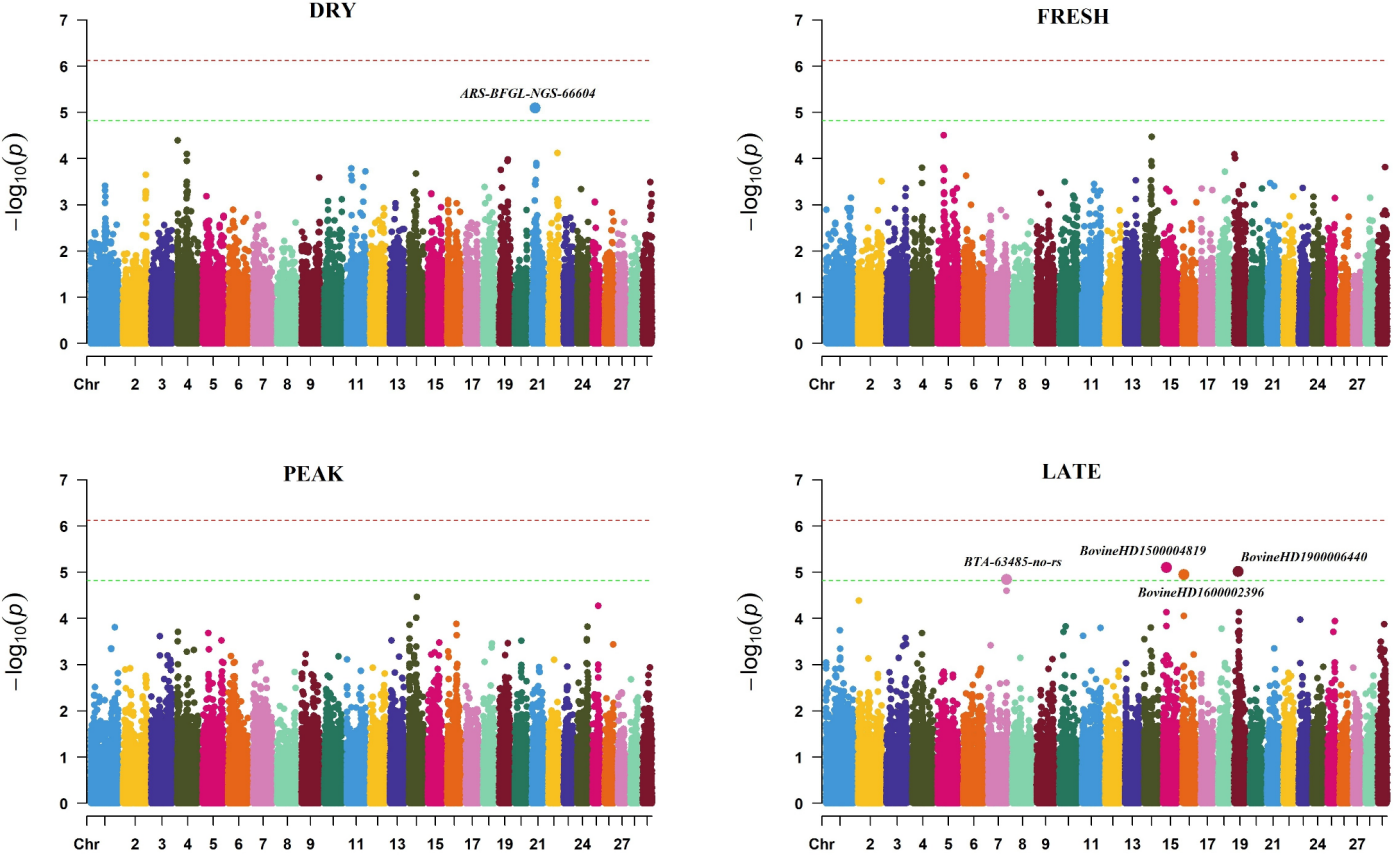
Manhattan plots illustrating the − log_10_(*P*-values) of single nucleotide polymorphisms across the 29 autosomes for the phenotype denoting presence or absence of digital dermatitis (BINP) recorded at four timepoints of foot examination (DRY: up to 120 days before calving, FRESH: 1–21 days after calving, PEAK: 50–120 days after calving and LATE: 170–305 days after calving). The red horizontal line represents the genome-wide significance threshold, while the green horizontal line denotes the suggestive significance threshold.

Figure 1 illustrates the profiles of the SNP P-values (expressed as − log_10_ values) for BINP at the four timepoints of foot examination. No single SNP reached genome-wide significance, but five suggestive significant SNPs were detected and are summarised in Supplementary Table S1. One marker on chromosome 21 was associated with DRY and four markers on chromosomes 7, 15, 16 and 19 were identified for LATE.

**Fig. 2:**
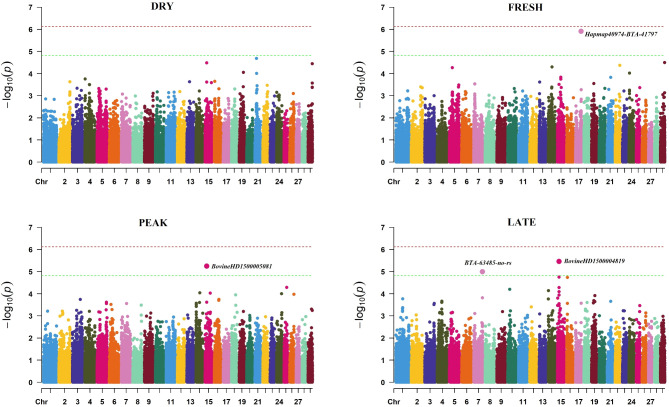
Manhattan plots showing the − log_10_(*P*-values) of SNPs across the 29 autosomal chromosomes the proportion of healthy feet phenotype (PROP) recorded at four timepoints of foot examination (DRY: up to 120 days before calving, FRESH: 1–21 days after calving, PEAK: 50–120 days after calving and LATE: 170–305 days after calving). The red horizontal line represents the genome-wide significance threshold, while the green horizontal line denotes the suggestive significance threshold.

Estimates of λ (0.99–1) and Q–Q plots indicate absence of inflation bias in the PROP analysis, too (Supplementary Fig. 2). Figure 2 illustrates the profiles of the SNP P-values (expressed as − log_10_ values) across the four timepoints of foot examination for PROP. Four suggestive significant SNPs were detected for FRESH, PEAK and LATE (Supplementary Table S2). Comparison of the SNP signals between BINP and PROP resulted in two common markers, namely BTA-63485-no-rs and BovineHD1500005081 that were located on chromosome 7 (103,114,045 bp) and 15 (19,773,230 bp), respectively.

Estimates of the genetic variance explained for the statistically significant SNPs were low for both DD phenotypes, ranging from 0.07 to 0.16% for BINP and from 0.05 to 0.18% for PROP (Supplementary Tables S1 and S2).

### Genomic window analysis

**Fig. 3:**
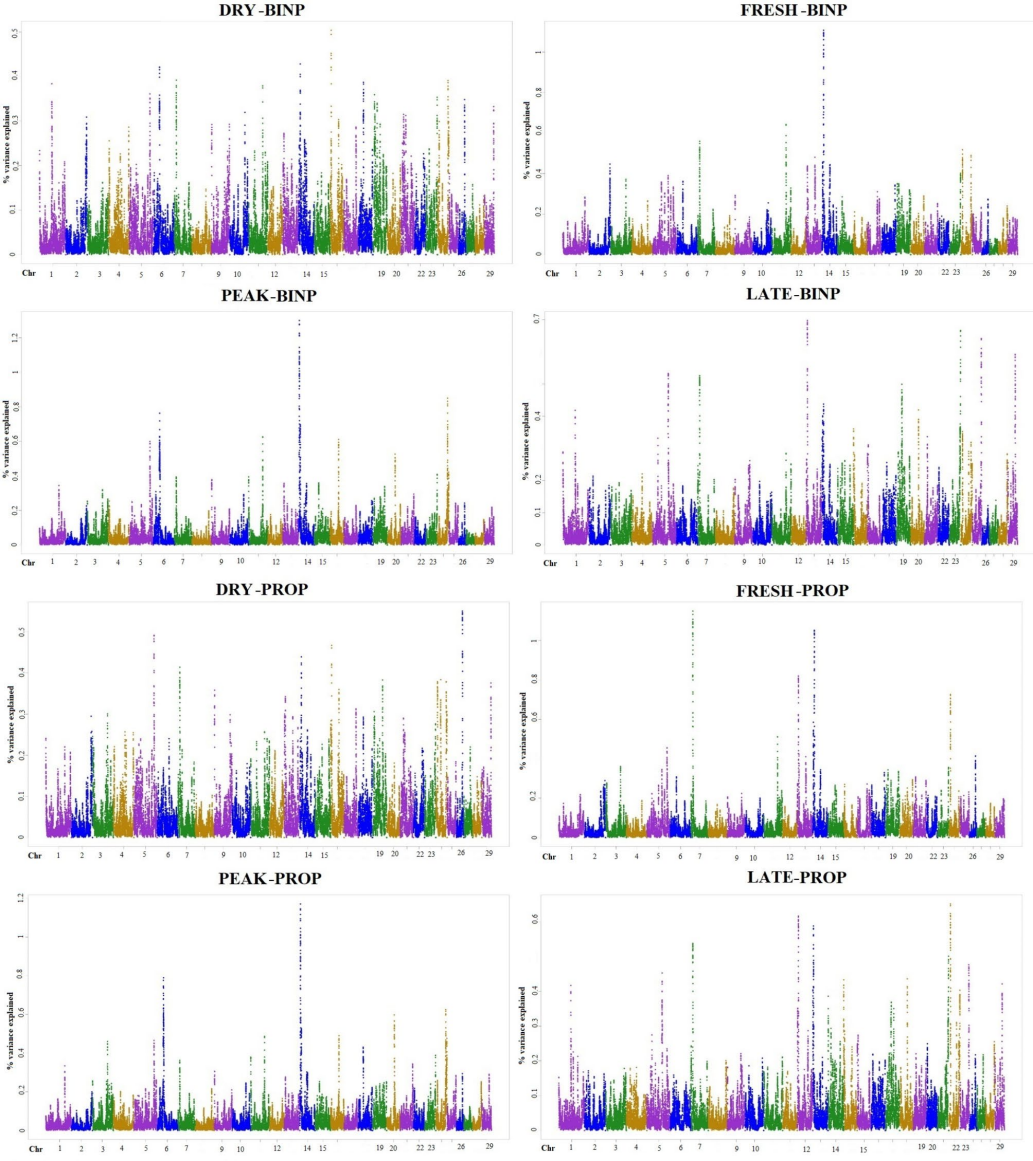
Manhattan plots of the genetic variance explained by 1 Mb sliding window for presence or absence of digital dermatitis (BINP) and proportion of healthy feet (PROP) phenotypes per timepoint of foot examination (DRY: up to 120 days before calving, FRESH: 1–21 days after calving, PEAK: 50–120 days after calving and LATE: 170–305 days after calving).

Figure 3 shows the Manhattan plots with respective percentages of genetic variance explained by 1-Mb windows per trait. A detailed description of the genomic windows that included the significant markers (Supplementary Tables S1 and S2) and the top ten genomic windows by proportion of the trait genetic variance explained is provided in Supplementary Table S3. The highest percentage of genetic variance was explained by a 1-Mb window on chromosome 14 (2,797,039–3,792,001 bp) and corresponded to 1.30% of the BINP variance at PEAK. The same region was also detected for PROP at PEAK explaining 1.17% of the total genetic variance. Three additional regions located on chromosomes 7 (12,261,707–13,253,152 bp) and 14 (5,821,914–6,816,219 bp and 5,998,335–6,962,216 bp) explained more than 1% of BINP and PROP variance at FRESH (Supplementary Table S3). Across traits, the top ten windows explained from 0.36 to 1.30% of the genetic variance while the genomic regions that included the significant SNPs explained from 0.10 to 0.27% of the genetic variance of the corresponding traits.

A total of 463 positional genes (Supplementary Table S3) were identified within the genomic windows and were considered in the ensuing functional enrichment analysis.

### Functional enrichment analysis

Functional enrichment analysis identified 29 significantly enriched Gene Ontology (GO) Biological Processes (BPs) and 73 corresponding genes (Table 2). These BPs included the lipoxygenase pathway (GO:0019372) with the *ALOXE3 (arachidonate lipoxygenase 3), ALOX12B (arachidonate 12-lipoxygenase, 12R type), ALOX12E (arachidonate lipoxygenase, epidermal), ALOX15 (arachidonate 15-lipoxygenase), and ALOX12 (arachidonate 12-lipoxygenase, 12S type)* genes, the negative regulation of interleukin-12 production (GO:0032695) with the *CMKLR1 (chemerin chemokine-like receptor 1), C1QBP (complement C1q binding protein), ARRB2 (arrestin beta 2)* genes, and the establishment of skin barrier (GO:0061436) with the *ALOXE3, ALOX12B, ALOX12* genes.

**Table 2:** Significantly enriched gene ontology (GO) biological process (BP) terms and the respective genes.

GO BP term	GO_ID	FDR P-value	Genes
Linoleic acid metabolic process	GO:0043651	9.07525E-06	*ALOXE3, ALOX12B, ALOX12E, ALOX15, ALOX12*
Hepoxilin biosynthetic process	GO:0051122	9.07525E-06	*ALOXE3, ALOX12B, ALOX12E, ALOX15, ALOX12*
Arachidonic acid metabolic process	GO:0019369	9.07525E-06	*ALOXE3, ALOX12B, ALOX12E, ALOX15, ALOX12, DAGLA*
Lipoxygenase pathway	GO:0019372	1.38377E-05	*ALOXE3, ALOX12B, ALOX12E, ALOX15, ALOX12*
Lipid oxidation	GO:0034440	0.000102575	*ALOX12B, ALOX12E, ALOX15, ALOX12*
Signal transduction	GO:0007165	0.001499945	*OR4D2G, CMKLR1, YWHAE, CXCL16, SCGB1A1, GRK3, GDNF, VAMP2, MC4R, GUCY2D, OR4D2B, GPR20, INS, CHRNE, RABEP1, OR4D2D, ADORA1, SST, OR4D1, GNG3, OR4D2, OR4D2E, IL10, ARRB2, IGF2, ABR*
Protein insertion into membrane	GO:0051205	0.001499945	*RTP2, RTP1, RTP4*
Negative regulation of interleukin-12 production	GO:0032695	0.002887765	*CMKLR1, C1QBP, ARRB2*
Glucose import in response to insulin stimulus	GO:0044381	0.002887765	*INS, TRARG1, SLC2A4*
Metanephros development	GO:0,001,656	0.009219858	*OSR1, GDNF, BCL2, IRX3*
Cell morphogenesis	GO:0000902	0.011206747	*CAPZB, CDH20, PDPN, BCL2, BCL6*
Protein insertion into ER membrane	GO:0045048	0.011773358	*GET3, CCDC47, WDR83OS*
RNA phosphodiester bond hydrolysis, endonucleolytic	GO:0090502	0.014714332	*FEN1, AGO2, RNASEH2A, RNASEK, NUDT12*
Lipoxin A4 biosynthetic process	GO:2001303	0.014714332	*ALOX15, ALOX12*
Detection of chemical stimulus involved in sensory perception of smell	GO:0050911	0.017112736	*OR4D2G, OR4D2B, OR4D2D, OR4D1, OR4D2, OR4D2E*
Cell–cell adhesion	GO:0098609	0.024088927	*CDH20, CDH2, LPP, PDPN, BCL2, DLG4*
Negative regulation of MDA-5 signalling pathway	GO:0039534	0.029858614	*TKFC, C1QBP*
DNA replication, removal of RNA primer	GO:0043137	0.029858614	*FEN1, RNASEH2A*
Carbohydrate metabolic process	GO:0005975	0.029858614	*MAN2B1, CHST12, SLC3A2, TKFC, INS, GANAB, CHI3L1, IGF2*
Anterior head development	GO:0097065	0.029858614	*PFAS, DDX10*
Negative regulation of extrinsic apoptotic signalling pathway in absence of ligand	GO:2001240	0.031215863	*GDNF, BCL2, PRDX2*
Detection of chemical stimulus involved in sensory perception of bitter taste	GO:0001580	0.031215863	*RTP2, RTP1, RTP4*
Establishment of skin barrier	GO:0061436	0.037628918	*ALOXE3, ALOX12B, ALOX12*
Sphingolipid metabolic process	GO:0006665	0.038985842	*ALOXE3, ALOX12B, FADS3, KDSR*
G protein-coupled receptor signalling pathway	GO:0007186	0.042280176	*OR4D2G, CMKLR1, GRK3, MC4R, OR4D2B, GPR20, PIK3R6, INS, OR4D2D, ADORA1, OR4D1, GNG3, OR4D2, OR4D2E*
Negative regulation of retrograde protein transport, ER to cytosol	GO:1904153	0.042280176	*DERL2, YOD1*
Neurotransmitter biosynthetic process	GO:0042136	0.042280176	*TH, DAGLA*
Ureteric bud development	GO:0001657	0.042280176	*OSR1, GDNF, BCL2*
Hydrogen peroxide catabolic process	GO:0042744	0.04501727	*MPO, LPO, PRDX2*

## Discussion

In the present study, we combined extensive phenotypic records on foot health with animal pedigree and genomic data to investigate the genetic background of DD in dairy cows. We explored the disease profile at four distinct stages of the lactation. In all cases both studied phenotypes, BINP and PROP, exhibited substantial levels of heritable genetic variance, underpinning the possibility to readily identify individuals with superior genetic profiles for resistance to DD development. The latter implies that the studied DD phenotypes would be amenable to genetic improvement through selective breeding.

We performed single-step genome-wide association (ssGWA) analyses, which simultaneously combine all genotypic, phenotypic and pedigree information available^13^. Our genomic heritability estimates for BINP (0.21–0.24) were slightly higher than Schöpke et al.^10^ (0.19), who investigated a similar binary trait but for only one observation period using data on 729 Holstein heifers in the USA. For PROP, our genomic heritability estimates (PROP: 0.21–0.25) were lower than Biemans et al.^11^ (0.37), who also examined the fraction of observations a cow was DD free by scoring 1513 Holstein–Friesian cows in the Netherlands. Other heritability estimates in previous studies^5,11,14^ fell within the wide range of 0.01–0.40, reflective of differences in data, populations, trait definition, and methods of statistical analysis. Nevertheless, most studies agreed that development of DD can be attributed to both genetic contributions and environmental influences.

Our single locus analyses highlighted six SNPs on autosomes 7, 15, 16, 17, 19 and 21 associated with the studied traits. Two of these markers on chromosomes 7 and 15 affected both DD phenotypes, namely BINP at the LATE stage and PROP at the PEAK and LATE stages of the lactation cycle. Despite reaching a suggestive statistical significance level after multiple testing adjustments, the proportion of the additive genetic variance of the trait accounted for by each SNP was below 0.18%, suggesting a likely polygenic control of the DD phenotypes of study.

According to Aguilar et al.^15^, a *p*-value determines if an individual marker has an effect that is seemingly different from zero with statistical assessment, while the actual SNP effect size mainly associates with the attributable part of the genetic variance of the trait, with no statistical assessment. Therefore, in the present study, we examined further the genomic regions harbouring the significant SNPs, together with the top 10 genomic windows by amount of variance explained.

For the window-based ssGWA, we utilised the sliding windows approach which has been proposed as a robust method to detect combined significant patterns of clustered SNPs associated with complex polygenic traits^16^. Two key points emerged from our results. Firstly, the top 10 genomic windows accounting for the highest proportion of genetic variance of the trait were located in multiple autosomes across the entire genome. Secondly, regions harbouring the suggestive significant individual SNPs from single locus ssGWA did not overlap with the top 10 genomic windows by proportion of genetic variance explained. These observations further attest to the polygenic nature of the studied phenotypes, where multiple genes controlling DD are widely distributed across the genome, each with a relatively modest effect. This profile fits the infinitesimal model assumptions for complex trait analyses.

The identified genomic windows of the present study harboured positional candidate genes, whose functional relevance to DD, was explored with functional enrichment analysis. Based on the results of this analysis, the *ALOXE3 (arachidonate lipoxygenase 3), ALOX12B (arachidonate 12-lipoxygenase, 12R type), ALOX12E (arachidonate lipoxygenase, epidermal), ALOX15 (arachidonate 15-lipoxygenase), ALOX12 (arachidonate 12-lipoxygenase, 12S type), CMKLR1 (chemerin chemokine-like receptor 1), C1QBP (complement C1q binding protein) and ARRB2 (arrestin beta 2)* genes could be associated with DD development. Among them, the *ALOXE3, ALOX12B, ALOX12E, ALOX15 and ALOX12* genes belong to the family of lipoxygenases, which play a known role in the modulation of epithelial proliferation and/or differentiation as well as in inflammation, wound healing, inflammatory skin diseases^17^. Indeed, the *ALOXE3* gene has been reported in a recent study of sole ulcer, a non-contagious hoof condition in cattle^18^. The latter study reported the same genomic region in chromosome 19 that was identified here, encompassing the *ALOXE3 (arachidonate lipoxygenase 3), HES7 (hes family bHLH transcription factor 7), U6 (U6 spliceosomal RNA), PER1 (period circadian regulator 1), VAMP2 (vesicle associated membrane protein 2), U8 (U8 small nucleolar RNA), TMEM107 (transmembrane protein 107), BORCS6 (BLOC-1 related complex subunit 6), AURKB (aurora kinase B), CTC1 (CST telomere replication complex component 1), PFAS (phosphoribosylformylglycinamidine synthase), RANGRF (RAN guanine nucleotide release factor), SLC25A35 (solute carrier family 25 member 35) and ARHGEF15 (Rho guanine nucleotide exchange factor 15)* genes.

Amongst the other genes identified in the present study, *CMKLR1* reportedly influences adipose tissue development, inflammation, and glucose homeostasis in mice^19^. In cattle, chemerin is a novel regulator of lactogenesis via its own receptor in mammary epithelial cells^20^. Moreover, *C1QBP* is involved in regulation of T cell proliferation^21^ and has been reportedly associated with milk protein percentage in cattle^22^. For *ARRB2,* a mouse study^23^ found that β-arrestin 2 inhibits the production of proinflammatory chemokines in cultured epidermal keratinocytes in response to T cell–derived cytokines in vitro and in T cell–driven allergic skin inflammation in vivo.

In addition to the aforementioned genes, the *DDX10 (DEAD-box helicase 10)* gene participating in the anterior head development has been previously associated with the foot-and-mouth disease in cattle^24^. A recent study^25^ reported functional variants in this gene potentially involved in innate immune response. Furthermore, six genes *(KHDRBS3, RBL2, PDPN, RTTN, BSND* and *LCORL*) reported here were also identified in a previous study on non-infectious claw horn disruptive lesions in the same cattle population, where *KHDRBS3 (KH RNA binding domain containing, signal transduction associated 3) and RBL2 (RB transcriptional corepressor like 2)* were associated with sole ulcer, *PDPN (podoplanin), RTTN (rotatin) and BSND (barttin CLCNK type accessory subunit beta)* with sole haemorrhage, and *LCORL (ligand dependent nuclear receptor corepressor like)* with white line disease^26^.

In the present study, DD phenotypes were defined at four distinct timepoints in a cow’s lactation. Each timepoint is associated with different metabolic and physiological state, conditions, and challenges for the animal. Certain overlap in suggestive significant SNPs (Supplementary Tables S1 and S2) and genomic windows (Supplementary Table S3) were observed across these timepoints. Moreover, genomic correlations between the DD phenotypes at different stages of lactation were sufficient high to imply a similar genomic profile controlling the disease development throughout the cow’s production cycle. Given the polygenic nature of the traits, the latter is the strongest result in this respect, suggesting that genetic selection to improve DD resistance at any timepoint would have an intertemporal benefit to foot health.

Instead of farmer or hoof trimming records used widely in previous studies^5,27^, we deployed here a large number of phenotypic records based on thorough inspections of lifted cow feet by a trained veterinarian. Although, admittedly, we involved a limited number of four farms, the focus of our study was on the accuracy and detail of individual clinical phenotyping for the in-depth analysis of DD.

In conclusion, results from the present study contribute to the understanding of the genetic mechanism underlying the DD lesion development in dairy cattle across distinct stages of the lactation cycle. Sizeable heritability estimates, combined with the largely polygenic mode of genetic control revealed in ssGWA analyses suggest that genomic selection based estimated breeding values would be the best approach to enhancing the inherent resistance of animals to DD infection. Genomic variants identified in the present study may be incorporated in and inform the genetic evaluation processes leading to increased accuracy of genomic selection. Our findings could contribute to future meta-analyses or multivariate analyses with larger samples size in order to detect genome-wide significant markers. Results may also serve as a basis for functional gene expression studies, aiming to identify causal genetic entities impacting on DD.

## Methods

### Ethics declarations

Ethical approval for the study was granted by the University of Liverpool Research Ethics Committee (VREC466ab, VREC269a). ASPA regulated procedures were conducted under a Home Office License (P191F589B). The study was conducted in accordance with the ARRIVE guidelines and relevant guidelines and regulations of the Journal.

### Data and quality control

Our data included 2,192 dairy cows from four farms in the UK. All animals were clinically examined by a veterinarian at four timepoints corresponding to distinct stages of the lactation: the pre-calving stage (DRY; up to 120 days before calving, mean = 55.3 days before calving), immediately after calving (FRESH; 0–21 days in milk, mean = 5.4 days in milk), near peak yield (PEAK; 50–120 days in milk, mean = 83.9 days in milk), and the latter part of lactation (LATE; 170–305 days in milk, mean = 199.6 days in milk). At each timepoint, every foot was examined separately for DD and was scored according to the M-stage system^28^, where M0 indicated healthy feet free of DD whereas the other values corresponded to different levels of infection. Supplementary Table S4 summarises these data.

Subsequently, two DD traits were defined for each timepoint: (i) A binary phenotype (BINP), where cows with a score other than M0 in at least one foot were considered cases and assigned the value of one and cows whose feet were all healthy (M0) were controls and assigned the value of zero; thus, BINP reflected the proportion of cows with DD at each timepoint of examination. (ii) A phenotype describing the proportion (PROP) of healthy (M0) feet of a cow. Data on the studied phenotypes are summarised in Table 3.

**Table 3:** Number of animals and descriptive statistics of the phenotypes per timepoint of foot examination.

Timepoint*	Number of animals	Percentage of infected animals (BINP)	Distribution of proportion of healthy feet (PROP; n = number of animals)
DRY	2031	28%	0.00 (n = 4), 0.25 (n = 23), 0.50 (n = 228), 0.75 (n = 309), 1.00 (n = 1,467)
FRESH	1945	24%	0.00 (n = 168), 0.25 (n = 1), 0.50 (n = 237), 0.75 (n = 61), 1.00 (n = 1,478)
PEAK	1890	19%	0.00 (n = 5), 0.25 (n = 5), 0.33 (n = 1), 0.50 (n = 143), 0.75 (n = 213), 1.00 (n = 1,523)
LATE	1716	20%	0.00 (n = 3), 0.25 (n = 6), 0.50 (n = 133), 0.67 (n = 1), 0.75 (n = 195), 1.00 (n = 1,378)

*DRY: up to 120 days before calving, FRESH: 1–21 days after calving, PEAK: 50–120 days after calving and LATE: 170–305 days after calving.

Genome-wide genotypes imputed to the 80 K Single Nucleotide Polymorphisms (SNP) panel used in the UK national dairy evaluations were available for all the studied animals^29^. Only autosomal SNPs were considered here. Quality control was performed firstly at sample and secondly at SNP marker level. We excluded animals with call rate lower than 0.90 and autosomal heterozygosity outside the 1.5 inter-quartile range. We also excluded SNPs with call rate lower than 0.90, minor allele frequency lower than 0.05 and those markers that deviated from Hardy–Weinberg equilibrium test P-value lower than 0.0001. Quality control was performed in PLINK^30^. After these filters, a total of 66,604 SNPs were retained and the number of animals retained were shown in Table 3.

A comprehensive, quality-assured pedigree dataset was also available for the study population, generated from the UK national genetic evaluation service (EGENES). A pedigree comprising 14,092 animals and spanning seven generations of the phenotyped animals was extracted and used in the ensuing data analyses.

### Principal component analysis

The genomic structure of the studied population was explored with a principal component (PC) analysis of the animals’ genotypes. The top 20 PCs were estimated with the ‘—pca’ command in PLINK^30^. The first three PCs were plotted on the two-dimensional space to assess genetic patterns in the studied population (Supplementary Fig. 3). The first PC was then included in the ensuing genomic analyses to account for these patterns.

### Single-step genome-wide association (ssGWA) analyses

The following univariate mixed model was applied for the analysis of each cow DD phenotype (BINP, PROP) at each timepoint of foot examination (DRY, FRESH, PEAK, LATE), separately, with the use of BLUPF90 family software^13,31^:

1 $${\text{y}} = {\text{W}}\upbeta + {\text{ Zg }} + {\text{ e}}$$

where y is a n × 1 vector of phenotypic values, β is a vector of fixed effects including farm, lactation number, calendar month of calving, age of cow at calving, and the first PC from the PC analysis of genotypes; g is the vector of the random animal additive genetic effects following var(g) ~ N(0, Hσ^2^_g_) distribution, where H is the matrix of additive genetic relationships incorporating both pedigree and genomic information and σ^2^_g_ is the additive genetic variance^32^; e a vector of random residuals with distribution of var(e) ~ N(0, I σ^2^_e_) in which I is the identity matrix and σ^2^_e_ is the residual variance; W and Z are incidence matrices relating records to β and g, respectively.

In order to combine all available data in ssGWA including both genotyped and ungenotyped animals the H matrix was generated in model (1) as follows^32–34^:

$$H^{ - 1} = \left[ {\begin{array}{*{20}c} 0 & 0 \\ {0 G^{ - 1} } & { - A_{22}^{ - 1} } \\ \end{array} } \right] + A^{ - 1}$$

where ${A}^{-1}$ is the inverse of the numerator pedigree relationship matrix encompassing all animals in the pedigree file, ${A}_{22}^{-1}$ is the inverse of the submatrix of *A* pertaining to the genotyped animals and ${G}^{-1}$ is the inverse of the genomic relationship matrix of the genotyped animals. The *G* matrix was constructed as proposed by VanRaden^35^.

Heritability of each trait at each timepoint was calculated as the ratio of the estimated additive genetic variance to the phenotypic variance from model (1).

BLUPF90+ was used to estimate genomic breeding values for each animal. SNP effects and P-values were then obtained by back-solving genomic breeding values using POSTGSF90^13,15^. The Bonferroni correction method was implemented to correct for multiple testing using R (http://www.r-project.org/). A SNP was considered significant at the genome-wide or suggestive level when the respective P-value was lower than 7.507057e-07(0.05/66,604) or 1.501411e-05 (1/66,604).

To characterise the extent to which the observed distribution of the test statistic followed the expected (null) distribution, quantile–quantile (Q–Q) plots were constructed. These plots in combination with estimates of the genomic inflation factor (λ) were used to assess potential systematic bias arising from population structure or the analytical approach^12^. The Manhattan and Q–Q plots were constructed using the CMplot package (https://github.com/YinLiLin/R-CMplot) in R (http://www.r-project.org/).

The proportion of genetic variance explained by a single significant SNP for each trait was estimated as follows^13^:

$$\frac{Var({Z}_{j}\widehat{{u}_{j}})}{{\sigma }_{g}^{2}}\times 100\%$$

where ${\sigma }_{g}^{2}$ is the total genetic variance, ${Z}_{j}$ is a vector of the gene content of the j^th^ SNP for all animals and $\widehat{{u}_{j}}$ is the estimated marker effect of the j^th^ SNP.

In addition to the single-locus analyses, a 1 Mb sliding-window approach was implemented using POSTGSF90 to calculate the corresponding proportion of genetic variance explained by each genomic window^36^. The Manhattan plots of the proportion of genetic variance (%) explained by each genomic window were constructed with the POSTGSF90 software.

Subsequently, a series of bivariate analyses were performed between different timepoints in the lactation for each phenotype using model (1). Genetic correlations were estimated between DRY, FRESH, PEAK and LATE for BINP and PROP using the BLUPF90 family software^13,31^.

### Positional candidate genes and functional enrichment analysis

Positional candidate genes were explored in the genomic windows that included significant SNPs from single locus ssGWA as well as the ten genomic windows that accounted for the highest proportion of the trait genetic variance. The latter aimed at regions harbouring multiple SNPs each with a small undetected effect that could collectively produce a discernible signal^37,38^. Positional candidate genes were retrieved from the Ensembl BioMarts hub^39^ based on the ARS-UCD1.2 bovine genome assembly.

All positional candidate genes were subjected to Gene Ontology (GO) Biological Process (BP) enrichment analysis using GeneCodis 4 (https://genecodis.genyo.es/,^40,41^). For functional enrichment analysis, we selected the species of *Bos taurus* for input genes and searched for GO BP significantly enriched annotations with FDR P-values lower than 0.05 according to the hypergeometric test.

## Electronic supplementary material

Below is the link to the electronic supplementary material.

Supplementary Material 4

Supplementary Material 5

Supplementary Material 6

Supplementary Material 4

Supplementary Material 5

Supplementary Material 6

Supplementary Material 7

## Data Availability

The data that support the findings of this study are available from Scotland’s Rural College (SRUC), but restrictions apply to certain data that are used under license for the current study. Data can become available from the corresponding author upon reasonable request and with permission of SRUC.
