# Supplementary figures and images for: Genome-wide association studies of dairy cattle resistance to digital dermatitis recorded at four distinct lactation stages
Source: Sci Rep. 2025 Mar 15;15:8922. doi: 10.1038/s41598-025-92162-x (PMC11909109; doi:10.1038/s41598-025-92162-x)

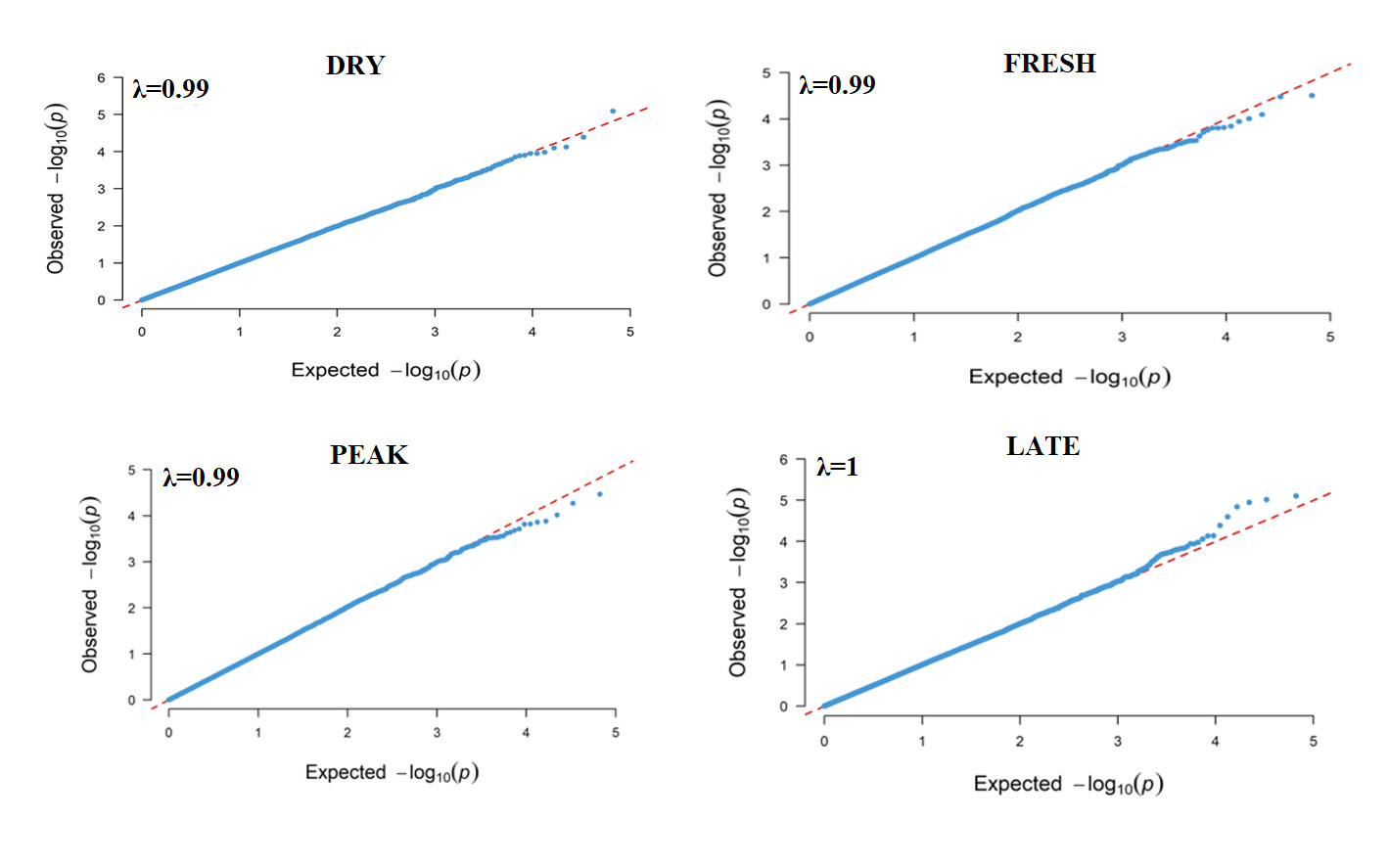

Supplement: Supplementary file 1 — Supplementary Material 4 [file 41598_2025_92162_MOESM1_ESM.jpg]

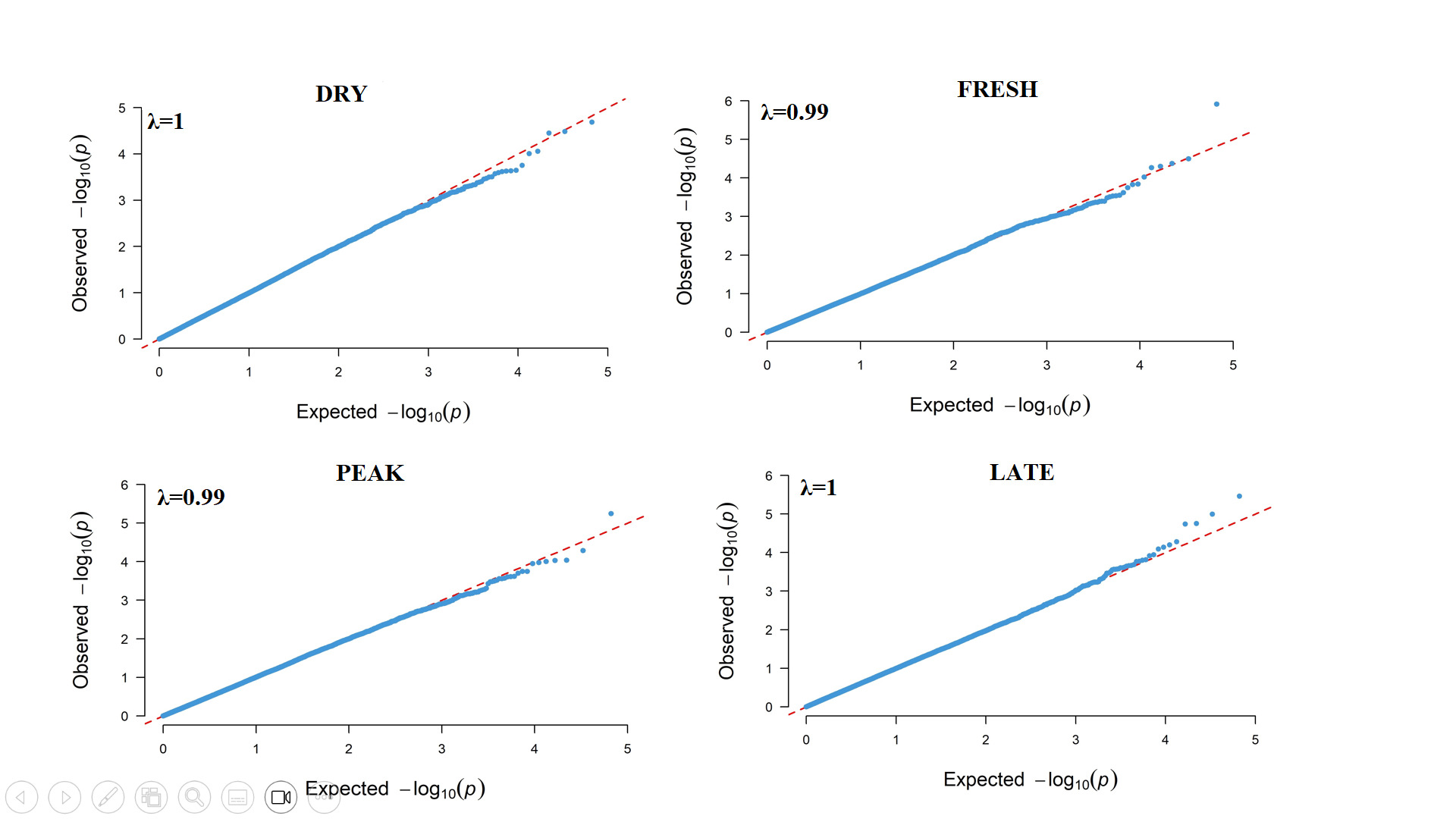

Supplement: Supplementary file 2 — Supplementary Material 5 [file 41598_2025_92162_MOESM2_ESM.jpg]

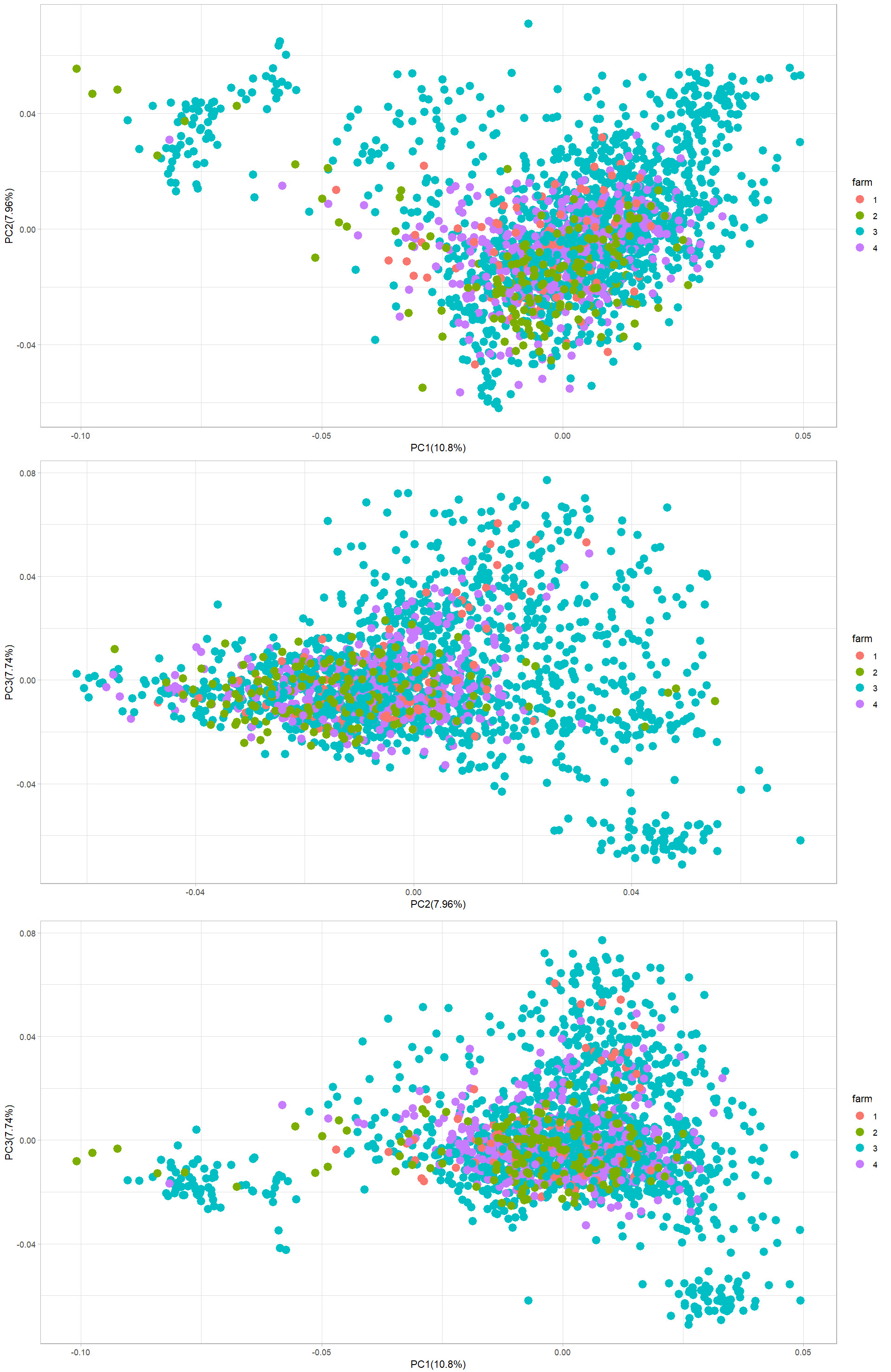

Supplement: Supplementary file 3 — Supplementary Material 6 [file 41598_2025_92162_MOESM3_ESM.jpg]
