# Supplementary material for: Genome-wide association studies of dairy cattle resistance to digital dermatitis recorded at four distinct lactation stages
Source: Sci Rep. 2025 Mar 15;15:8922. doi: 10.1038/s41598-025-92162-x (PMC11909109; doi:10.1038/s41598-025-92162-x)
Supplement: Supplementary file 4 — Supplementary Material 4 [file 41598_2025_92162_MOESM4_ESM.pdf]

Table S1. Suggestive significant markers for the binary (presence or absence of digital dermatitis) phenotype.

**SNP**	**Chromosome**	**Position (bp)**	**P-value**	**Genetic variance explained**	**Timepoint of foot examination**
ARS-BFGL-NGS-66604	21	18,496,366	8.09E-06	0.097	DRY (up to 120 days before calving)
BTA-63485-no-rs	7	103,114,045	1.44E-05	0.162	LATE (170-305 days after calving)
BovineHD1500005081	15	19,773,230	7.88E-06	0.091	LATE (170-305 days after calving)
BovineHD1600002396	16	8,011,283	1.13E-05	0.104	LATE (170-305 days after calving)
BovineHD1900006440	19	21,981,558	9.66E-06	0.072	LATE (170-305 days after calving)
