# Supplementary material for: Genome-wide association studies of dairy cattle resistance to digital dermatitis recorded at four distinct lactation stages
Source: Sci Rep. 2025 Mar 15;15:8922. doi: 10.1038/s41598-025-92162-x (PMC11909109; doi:10.1038/s41598-025-92162-x)
Supplement: Supplementary file 5 — Supplementary Material 5 [file 41598_2025_92162_MOESM5_ESM.pdf]

Table S2. Suggestive significant markers for the proportion of healthy feet (PROP) phenotype.

SNP	Chromosome	Position (bp)	P-value	Genetic variance explained	Timepoint of foot examination
Hapmap40974-BTA-41797	17	64,673,392	1.23E-06	0.146	FRESH (1-21 days after calving)
BovineHD1500005081	15	19,773,230	5.69E-06	0.049	PEAK (50-120 days after calving)
BTA-63485-no-rs	7	103,114,045	1.02E-05	0.176	LATE(170-305 days after calving)
BovineHD1500004819	15	18,672,450	3.48E-06	0.105	LATE(170-305 days after calving)
