# Supplementary material for: Genome-wide association studies of dairy cattle resistance to digital dermatitis recorded at four distinct lactation stages
Source: Sci Rep. 2025 Mar 15;15:8922. doi: 10.1038/s41598-025-92162-x (PMC11909109; doi:10.1038/s41598-025-92162-x)
Supplement: Supplementary file 6 — Supplementary Material 6 [file 41598_2025_92162_MOESM6_ESM.pdf]

Table S3. Top ten genomic windows with the highest percentage of genetic variance explained per trait and the genomic windows that included the significant markers.
The positional candidate genes are also given per genomic window.

Trait (timepoint*)	Chromosome	Genomic window in bp	Proportion of genetic variance explained(%)	Positional candidate genes within the genomic window
BINP (DRY)	16	4154168_5151533	0.50356	*SRGAP2,IKBKE,RASSFS,EIF2D,DYRK3,MAPKAPK2,IL10,IL19,IL20,IL24,FCMR,PIGR,FCAMR,C16H1orf116,YOD1,PFKFB2,C4BPB,C4BPA*
BINP (DRY)	14	5922777_6912869	0.4279	*KHDRBS3*
BINP (DRY)	6	37029371_38028236	0.42088	*LAP3,MED28,FAM184B,NCAPG,DCAF16,LCORL*
BINP (DRY)	7	12261707_13253152	0.39171	*CACNA1A,bta-mir-12000,IER2,STX10,NACC1,TRMT1,LYL1,NFIX,GADD45GIP1,RAD23A,CALR,FARSA,SYCE2,GCDH,KLF1,DNASE2,MAST1,RTBDN,RNASEH2A,PRDX2,JUNB,HOOK2,BEST2,GET3,TRIR,TNPO2,FBXW9,GNG14,DHPS,WDR83,WDR83OS,MAN2B1,bta-mir-12035*
BINP (DRY)	24	58877604_59845638	0.39065	*MC4R*
BINP (DRY)	18	21698312_22683964	0.38685	*CHD9,RBL2,AKTIP,RPGRIP1L,FTO,5S_rRNA,IRX3*
BINP (DRY)	1	79111744_80108636	0.38363	*LPP,BCL6,RTP2,SST,RTP4,MASP1,RTP1*
BINP (DRY)	11	78444403_79407746	0.37873	*PUM2,U6,SDC1,LAPTM4A,MATN3,WDR35,TTC32,OSR1*
BINP (DRY)	5	106066646_107053586	0.36076	*PARP11,CRACR2A,PRMT8,TSPAN11,TSPAN9,TEAD4,TULP3,RHNO1,FOXM1,TEX52,NRIP2,ITFG2,FKBP4,DDX11,WASHC1,IQSEC3*

BINP (DRY)	19	8367258 _9365637	0.35898	*MSI2,bta-mir-378d,CCDC182,MRPS23,CUEDC1,VEZF1,SRSF1,DYNLL2,OR4D2B,OR4D1B,OR4D2,OR4D1,OR4D2G,OR4D2E,OR4D2D,OR4D2F,MKS1,LPO,U6,MPO,U6,TSPOAP1,bta-mir-142,SUPT4H1,RNF43*
BINP (FRESH)	14	5821914 _6816219	1.1076	*KHDRBS3*
BINP (FRESH)	11	78402914 _79395085	0.64211	*PUM2,U6,SDC1,LAPTM4A,MATN3,WDR35,TTC32*
BINP (FRESH)	7	12261707 _13253152	0.55965	*CACNA1A,bta-mir-12000,IER2,STX10,NACC1,TRMT1,LYL1,NFIX,GADD45GIP1,RAD23A,CALR,FARSA,SYCE2,GCDH,KLF1,DNASE2,MAST1,RTBDN,RNASEH2A,PRDX2,JUNB,HOOK2,BEST2,GET3,TRIR,TNPO2,FBXW9,GNG14,DHPS,WDR83,WDR83OS,MAN2B1,bta-mir-12035*
BINP (FRESH)	24	6108827 _7100407	0.51776	*SOCS6*
BINP (FRESH)	24	57486568 _58477861	0.49007	*NEDD4L,bta-mir-122,ALPK2,MALT1,ZNF532,OACYL,SEC11C,GRP,RAX,CPLX4,LMAN1,CCBE1*
BINP (FRESH)	13	45500844 _46483918	0.48084	*ADARB2,WDR37,IDI1,GTPBP4,U6,LARP4B*
BINP (FRESH)	14	2797039 _3792001	0.45713	*PTK2,MIR151A,AGO2,CHRAC1,TRAPPC9,5S_rRNA,bta-mir-12027,KCNK9*
BINP (FRESH)	2	133011945 _134003964	0.44695	*TMCO4,HTR6,NBL1,MICOS10,U6,CAPZB,SLC66A1,AKR7A2,MRTO4,EMC1,SNORA70,UBR4,U3,U1,IFFO2,ALDH4A1,TAS1R2,PAX7*
BINP (FRESH)	14	41999758 _42992294	0.44421	*STMN2,HEY1*
BINP (FRESH)	13	5044816 _6038638	0.43777	*BTBD3,SNORA70*

BINP (PEAK)	14	2797039 _3792001	1.30007	*PTK2,MIR151A,AGO2,CHRAC 1,TRAPPC9,5S_rRNA,bta-mir-12027,KCNK9*
BINP (PEAK)	24	57486568 _58477861	0.85047	*NEDD4L,bta-mir-122,ALPK2,MALT1,ZNF532,OA CYL,SEC11C,GRP,RAX,CPLX4, LMAN1,CCBE1*
BINP (PEAK)	6	37412062 _38408158	0.76332	*LCORL*
BINP (PEAK)	14	5922777 _6912869	0.69689	*KHDRBS3*
BINP (PEAK)	11	78444403 _79407746	0.62625	*PUM2,U6,SDC1,LAPTM4A,MA TN3,WDR35,TTC32,OSR1*
BINP (PEAK)	16	53556874 _54545304	0.61122	*bta-mir-2285cv-2,PRDM2,PDPN,LRRC38*
BINP (PEAK)	5	106119381 _107114180	0.59892	*PARP11,CRACR2A,PRMT8,TS PAN11,TSPAN9,TEAD4, TULP3,RHNO1,FOXM1,TEX52, NRIP2,ITFG2,FKBP4,DDX11, WASHC1,IQSEC3*
BINP (PEAK)	20	35947127 _36934725	0.52602	*LIFR,EGFLAM,U6,U6,GDNF, WDR70*
BINP (PEAK)	24	60784574 _61778720	0.4213	*TNFRSF11A,ZCCHC2,PHLPP1 ,BCL2,KDSR,U6,VPS4B,SERPI NB5*
BINP (PEAK)	23	51353257 _52350972	0.40863	*GMDS,FOXF2,FOXQ1,5S_rRN A,EXOC2,IRF4,DUSP22*
BINP(LATE)	13	5044816 _6038638	0.69683	*BTBD3,SNORA70*
BINP(LATE)	23	51353257 _52350972	0.66585	*GMDS,FOXF2,FOXQ1,5S_rRN A,EXOC2,IRF4,DUSP22*
BINP(LATE)	25	39845396 _40831336	0.64079	*SDK1,bta-mir-2390,CARD11,GNA12,AMZ1,B RAT1,bta-mir-11980,IQCE,TTYH3,LFNG,bta-mir-12029,GRIFIN,CHST12*

BINP(LATE)	29	39803454 _40803159	0.59181	*VWCE,DDB1,TKFC,CYB561A3, TMEM138,TMEM216,CPSF7,SDHAF2,bta-mir-2405,PPP1R32,LRRC10B,SYT7, bta-mir-2885,DAGLA,MYRF,TMEM258, FEN1,FADS1,FADS2,FADS3,RAB3IL1,BEST1,FTH1,INCENP, SCGB1D,SCGB2A2,ASRGL1,SCGB1A1*
BINP(LATE)	5	85407207 _86395589	0.53231	*5S_rRNA,SOX5,7SK*
BINP(LATE)	7	12114348 _13098628	0.52564	*CACNA1A,bta-mir-12000,IER2,STX10,NACC1,TRMT1,LYL1,NFIX,GADD45GIP1, RAD23A,CALR,FARSA,SYCE2, GCDH,KLF1,DNASE2,MAST1, RTBDN,RNASEH2A,PRDX2,JUNB,HOOK2,BEST2,GET3,TRIR, TNPO2,FBXW9,GNG14,DHPS, WDR83,WDR83OS,MAN2B1*
BINP (LATE)	19	26012592 _27011004	0.49904	*NLRP1,MIS12,DERL2,DHX33, C1QBP,RPAIN,NUP88,bta-mir-199c,RABEP1,SCIMP,ZFP3,KIF1C,INCA1,CAMTA2,SPAG7,ENO3,PFN1,RNF167,SLC25A11, GP1BA,CHRNE,C19H17orf107, MINK1,PLD2,PSMB6,GLTPD2, VMO1,TM4SF5,ZMYND15,CXCL16,MED11,PELP1,ARRB2,bta-mir-2338,ALOX15,ALOX12E,ALOX12,RNASEK,C19H17orf49,bta-mir-195,bta-mir-497,BCL6B,SLC16A13,SLC16A11,CLEC10A,ASGR2,ASGR1,DLG4,ACADVL,bta-mir-324,DVL2,PHF23,GABARAP,CTDNEP1,ELP5,CLDN7,SLC2A4,YBX2*

BINP (LATE)	19	27421507 _28417009	0.46117	*DNAH2,KDM6B,TMEM88,NAA38,CYB5D1,CHD3,RNF227,KCNAB3,TRAPPC1,CNTROB,GUCY2D,ALOX15B,ALOX12B,ALOXE3,HES7,U6,PER1,VAMP2,U8,TMEM107,BORCS6,AURKB,CTC1,PFAS,RANGRF,SLC25A35,ARHGEF15,ODF4,KRBA2,RPL26,RNF222,NDEL1,MYH10,U6,CCDC42,MFSD6L,PIK3R6,PIK3R5,bta-mir-2284aa-3*
BINP (LATE)	14	5922777 _6912869	0.43744	*KHDRBS3*
BINP (LATE)	20	35947127 _36934725	0.4192	*LIFR,EGFLAM,U6,GDNF,WDR70*
PROP (DRY)	26	44550619 _45536398	0.55027	*CTBP2,U6,TEX36,EDRF1,UROS,BCCIP,DHX32,U6,FANK1,ADAM12*
PROP (DRY)	5	106170583 _107114180	0.49133	*PARP11,CRACR2A,PRMT8,TSPAN11,TSPAN9,TEAD4, TULP3,RHNO1,FOXM1,TEX52, NRIP2,ITFG2,FKBP4,DDX11, WASHC1,IQSEC3*
PROP (DRY)	16	4154168 _5151533	0.46703	*SRGAP2,IKBKE,RASSFS,EIF2D,DYRK3,MAPKAPK2,IL10,IL19,IL20,IL24,FCMR,PIGR,FCAMR,C16H1orf116,YOD1,PFKFB2,C4BPB,C4BPA*
PROP (DRY)	14	5922777 _6912869	0.43896	*KHDRBS3*
PROP (DRY)	7	12261707 _13253152	0.4138	*CACNA1A,bta-mir-12000,IER2,STX10,NACC1,TRMT1,LYL1,NFIX,GADD45GIP1, RAD23A,CALR,FARSA,SYCE2, GCDH,KLF1,DNASE2,MAST1, RTBDN,RNASEH2A,PRDX2,JUNB,HOOK2,BEST2,GET3,TRIR, TNPO2,FBXW9,GNG14,DHPS, WDR83,WDR83OS,MAN2B1,bta-mir-12035*

PROP (DRY)	24	28638911 _29593728	0.38359	*CDH2*
PROP (DRY)	19	47056257 _48036082	0.38278	*MRC2,MARCHF10,TANC2,CYB561,ACE,KCNH6,DCAF7,TACO1,MAP3K3,LIMD2,STRADA,CCDC47*
PROP (DRY)	24	6070707 _7065050	0.37862	*SOCS6*
PROP (DRY)	24	58971639 _59971040	0.3786	*MC4R,CDH20*
PROP (DRY)	29	48454620 _49425799	0.37548	*OSBPL5,U6,CARS1,NAP1L4,PHLDA2,SLC22A18,CDKN1C,KCNQ1,TRPM5,TSSC4,CD81,TSPAN32,ASCL2,TH,INS,IGF2*
PROP (FRESH)	7	12261707 _13253152	1.14961	*CACNA1A,bta-mir-12000,IER2,STX10,NACC1,TRMT1,LYL1,NFIX,GADD45GIP1,RAD23A,CALR,FARSA,SYCE2,GCDH,KLF1,DNASE2,MAST1,RTBDN,RNASEH2A,PRDX2,JUNB,HOOK2,BEST2,GET3,TRIR,TNPO2,FBXW9,GNG14,DHPS,WDR83,WDR83OS,MAN2B1,bta-mir-12035*
PROP (FRESH)	14	5998335 _6962216	1.05208	*KHDRBS3*
PROP (FRESH)	13	4799708 _5797777	0.81975	*BTBD3,SNORA70*
PROP (FRESH)	24	6333278 _7316046	0.72579	*SOCS6,RTTN,CD226*
PROP (FRESH)	14	2606051 _3589056	0.585	*GPR20,SLC45A4,DENND3,PTK2,MIR151A,AGO2,CHRAC1,TRAPPC9,5S_rRNA,bta-mir-12027*
PROP (FRESH)	11	78402914 _79395085	0.51146	*PUM2,U6,SDC1,LAPTM4A,MATN3,WDR35,TTC32*
PROP (FRESH)	5	106170583 _107114180	0.45529	*PARP11,CRACR2A,PRMT8,TSPAN11,TSPAN9,TEAD4,TULP3,RHNO1,FOXM1,TEX52,NRIP2,ITFG2,FKBP4,DDX11,WASHC1,IQSEC3*

PROP (FRESH)	13	8748439 _9734692	0.42279	*MACROD2*
PROP (FRESH)	26	44966836 _45916519	0.41422	*U6,TEX36,EDRF1,UROS,BCCIP,DHX32,U6,FANK1,ADAM12*
PROP (FRESH)	13	45500844 _46483918	0.4102	*ADARB2,WDR37,IDI1,GTPBP4,U6,LARP4B*
PROP (PEAK)	14	2797039 _3792001	1.17066	*PTK2,MIR151A,AGO2,CHRAC1,TRAPPC9,5S_rRNA,bta-mir-12027,KCNK9*
PROP (PEAK)	6	37412062 _38408158	0.78938	*LCORL*
PROP (PEAK)	24	57486568 _58477861	0.62456	*NEDD4L,bta-mir-122,ALPK2,MALT1,ZNF532,OACYL,SEC11C,GRP,RAX,CPLX4,LMAN1,CCBE1*
PROP (PEAK)	20	35947127 _36934725	0.597	*LIFR,EGFLAM,U6,GDNF,WDR70*
PROP (PEAK)	14	5821914 _6816219	0.52691	*KHDRBS3*
PROP (PEAK)	16	53796504 _54785177	0.48987	*PRDM2,PDPN,LRRC38,CFAP107,AADACL3*
PROP (PEAK)	11	78444403 _79407746	0.4862	*PUM2,U6,SDC1,LAPTM4A,MATN3,WDR35,TTC32,OSR1*
PROP (PEAK)	24	60784574 _61778720	0.4762	*TNFRSF11A,ZCCHC2,PHLPP1,BCL2,KDSR,U6,VPS4B,SERPINB5*
PROP (PEAK)	5	106119381 _107114180	0.4653	*PARP11,CRACR2A,PRMT8,TSPAN11,TSPAN9,TEAD4, TULP3,RHNO1,FOXM1,TEX52, NRIP2,ITFG2,FKBP4,DDX11, WASHC1,IQSEC3*
PROP (PEAK)	3	90558078 _91549234	0.45992	*U6,USP24,BSND,TMEM61,DHCR24,LEXM,TTC22,PARS2,TTC4*
PROP (LATE)	24	6333278 _7316046	0.65115	*SOCS6,RTTN,CD226*
PROP (LATE)	13	4811787 _5797777	0.61649	*BTBD3,SNORA70*
PROP (LATE)	14	2797039 _3792001	0.58789	*PTK2,MIR151A,AGO2,CHRAC1,TRAPPC9,5S_rRNA,bta-mir-12027,KCNK9*

PROP (LATE)	7	12299051_13283360	0.5378	*CACNA1A,bta-mir-12000,IER2,STX10,NACC1,TRMT1,LYL1,NFIX,GADD45GIP1,RAD23A,CALR,FARSA,SYCE2,GCDH,KLF1,DNASE2,MAST1,RTBDN,RNASEH2A,PRDX2,JUNB,HOOK2,BEST2,GET3,TRIR,TNPO2,FBXW9,GNG14,DHPS,WDR83,WDR83OS,MAN2B1,bta-mir-12035*
PROP (LATE)	23	51353257_52350972	0.50108	*GMDS,FOXF2,FOXQ1,5S_rRNA,EXOC2,IRF4,DUSP22*
PROP (LATE)	25	39317822_40317729	0.47648	*SDK1,bta-mir-2390*
PROP (LATE)	5	85725559_86708225	0.45273	*SOX5,7SK*
PROP (LATE)	20	35947127_36934725	0.43606	*LIFR,EGFLAM,U6,GDNF,WDR70*
PROP (LATE)	16	875872_1873661	0.43286	*ADORA1,MYBPH,CHI3L1,BTG2,FMOD,PRELP,OPTC,U6,ATP2B4,LAX1,ZC3H11A,SNRPE*
PROP (LATE)	29	40229754_41193917	0.42114	*MYRF,TMEM258,FEN1,FADS1,FADS2,FADS3,RAB3IL1,BEST1,FTH1,INCENP,SCGB1D,SCGB2A2,ASRGL1,SCGB1A1,AHNAK,EEF1G,TUT1,MTA2,EML3,ROM1,B3GAT3,GANAB,INTS5,C29H11orf98,CSKMT,SNORA57,UQCC3,UBXN1,LRRN4CL,BSCL2,GNG3,HNRNPUL2,TTC9C,ZBTB3,POLR2G,TAF6L,TMEM179B,TMEM223,NXF1,STX5,WDR74,U2,SNORD22,SNORD31,SNORD30,SNORD29,SNORD22,SNORD28,SNORD27,SNORD26,SNORD25,SLC3A2*
BINP (DRY)	21	17499956_18496366	0.27	*AGBL1*
BINP (LATE)	16	8011283_8975307	0.1	*none*

BINP (LATE)	19	21593057 _22562168	0.12	*TRARG1,BHLHA9,U6,ABR,TIMM22,NXN,MRM3,GLOD4,bta-mir-2335,GEMIN4,DBIL5,TLCD3A,VPS53,bta-mir-2336,RFLNB,C19H17orf97,5S_rRNA,RPH3AL,DOC2B,YWHAE*
PROP (FRESH)	17	64534781 _65510040	0.2	*CMKLR1,WSCD2,bta-mir-6521,PIWIL3,SGSM1,LHFPL7,KIAA1671,7SK,CRYBB3,CRYBB2,GRK3*
PROP (PEAK)	15	18789880 _19775810	0.1	*C15H11orf87*
PROP (LATE)	7	102501837 _103472710	0.2	*7SK,NUDT12,U6*
PROP (LATE)	15	17907803 _18882017	0.17	*ATM,5S_rRNA,C15H11orf65,POGLUT3,EXPH5,DDX10*

*BINP: presence/absence of digital dermatitis, PROP: proportion of healthy feet ,
DRY: up to 120 days before calving, FRESH: 1-21 days after calving,
PEAK: 50-120 days after calving and LATE: 170-305 days after calving
