# Supplementary material for: Genome-wide association studies of dairy cattle resistance to digital dermatitis recorded at four distinct lactation stages
Source: Sci Rep. 2025 Mar 15;15:8922. doi: 10.1038/s41598-025-92162-x (PMC11909109; doi:10.1038/s41598-025-92162-x)
Supplement: Supplementary file 7 — Supplementary Material 7 [file 41598_2025_92162_MOESM7_ESM.pdf]

Supplementary Table S4. Percentage of cow feet by M-stage class* per timepoint of examination

M-stage class	Percentage of feet (DRY)	Percentage of feet (FRESH)	Percentage of feet (PEAK)	Percentage of feet (LATE)
M0	89.54%	56.41%	92.67%	91.67%
M1	0.38%	0.76%	0.41%	0.36%
M2	0.70%	1.04%	0.95%	0.74%
M3	0.55%	1.05%	1.30%	2.19%
M4	8.27%	5.57%	4.14%	3.57%
M4.1	0.55%	0.33%	0.29%	0.28%
Missing	NA	34.83%	0.24%	1.19%

* Döpfer, D. et al. Histological and bacteriological evaluation of digital dermatitis in cattle, with special reference to spirochaetes and Campylobacter faecalis. Vet. Rec. 140, 620–623
